# IPC 2.0: prediction of isoelectric point and p*K*_a_ dissociation constants

**DOI:** 10.1093/nar/gkab295

**Published:** 2021-04-27

**Authors:** Lukasz Pawel Kozlowski

**Affiliations:** Institute of Informatics, Faculty of Mathematics, Informatics, and Mechanics, University of Warsaw, Warsaw, Mazovian Voivodeship 02-097, Poland

## Abstract

The isoelectric point is the pH at which a particular molecule is electrically neutral due to the equilibrium of positive and negative charges. In proteins and peptides, this depends on the dissociation constant (p*K*_a_) of charged groups of seven amino acids and NH^+^ and COO^−^ groups at polypeptide termini. Information regarding isoelectric point and p*K*_a_ is extensively used in two-dimensional gel electrophoresis (2D-PAGE), capillary isoelectric focusing (cIEF), crystallisation, and mass spectrometry. Therefore, there is a strong need for the *in silico* prediction of isoelectric point and p*K*_a_ values. In this paper, I present Isoelectric Point Calculator 2.0 (IPC 2.0), a web server for the prediction of isoelectric points and p*K*_a_ values using a mixture of deep learning and support vector regression models. The prediction accuracy (RMSD) of IPC 2.0 for proteins and peptides outperforms previous algorithms: 0.848 versus 0.868 and 0.222 versus 0.405, respectively. Moreover, the IPC 2.0 prediction of p*K*_a_ using sequence information alone was better than the prediction from structure-based methods (0.576 versus 0.826) and a few folds faster. The IPC 2.0 webserver is freely available at www.ipc2-isoelectric-point.org

## Graphical Abstract

**Graphical Abstract:**
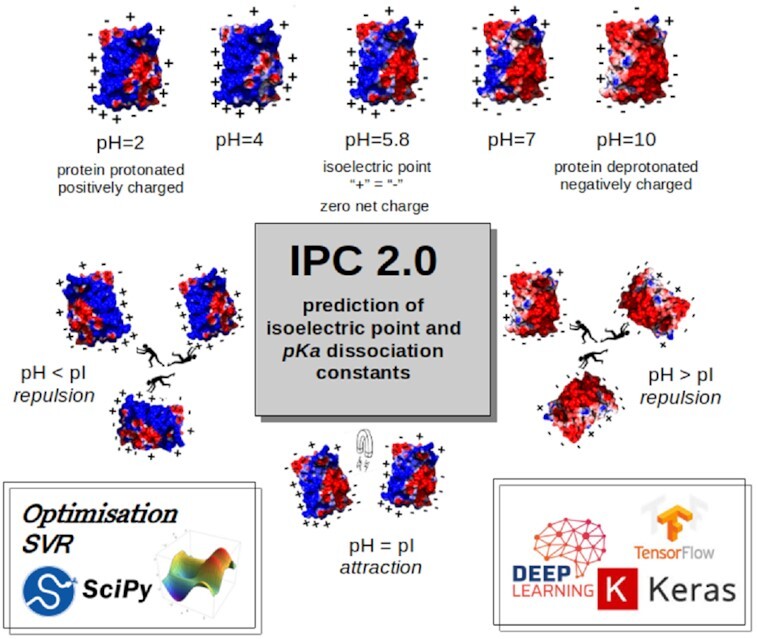
Isoelectric Point Calculator 2.0 - prediction of IEP with deep learning.

## INTRODUCTION

The isoelectric point (*pI* or *IEP*) is an important physicochemical parameter of many compounds, including peptides and proteins, and can be used to estimate the surface charge of molecules in various pH conditions. This physicochemical property has been extensively used in many molecular techniques, such as two-dimensional gel electrophoresis (2D-PAGE) (1,2), capillary isoelectric focusing (3,4), crystallisation (5), and mass spectrometry (MS) (6,7). It should be stressed that for polypeptides, the isoelectric point depends mostly on the acid dissociation constants (p*K*_a_) of the ionisable groups of seven charged amino acids: glutamate (γ-carboxyl group), cysteine (thiol group), aspartate (ß-carboxyl group), tyrosine (phenol group), lysine (ϵ-ammonium group), histidine (imidazole side chains), and arginine (guanidinium group). Furthermore, other charged groups can be important, such as the amine and carboxyl-terminal groups of the polypeptide chain and the post-translational modifications (PTMs) that carry the charged groups (e.g. phosphorylation and N-terminal acetylation). Moreover, the difference between the theoretical p*I* and the experimental p*I* can be related to the ionisation state of the individual residues. Some residues are buried inside the protein structure and, therefore, their contribution to the net charge of the whole molecule is marginal. Additionally, the charge of the exposed residue can be neutralised if it is used to form interactions with other residues, such as in non-covalent salt bridges, in which a proton migrates from a carboxylic acid group to a primary amine or to the guanidine group in Arg (in proteins, Lys or Arg are used as the bases and Asp or Glu as the acids; 8–10). However, most of the *in silico* methods that are currently used for p*I* estimation are based on simply counting the numbers of charged residues and utilising the Henderson-Hasselbalch equation with customised p*K*_a_ values (11,12). Nevertheless, some attempts to build more sophisticated methods should be acknowledged, such as those using genetic algorithms (13), artificial neural networks (14) and support vector machines (15).

While the estimation of the isoelectric point can be considered a challenging task, the prediction of p*K*_a_ values for individual residues is even more difficult. The average p*K*_a_ values used for p*I* prediction have been measured using simplified conditions, such as alanine pentapeptides with charged residue in the centre (16). This has been done to minimise the contribution from neighbouring residues, but such an approach is of no use for p*K*_a_ estimation in real proteins, where the influence of surrounding residues must be considered. An additional problem involved in building a reliable p*K*_a_ prediction algorithm is the scarcity of data (approximately 1000 known p*K*_a_ values in proteins have been measured experimentally (17)). To date, for p*K*_a_ prediction, only programs based on protein structure have been available, such as MCCE (18), H++ (19), Propka (20) and Rosetta p*K*_a_ (21).

**Figure 1.**
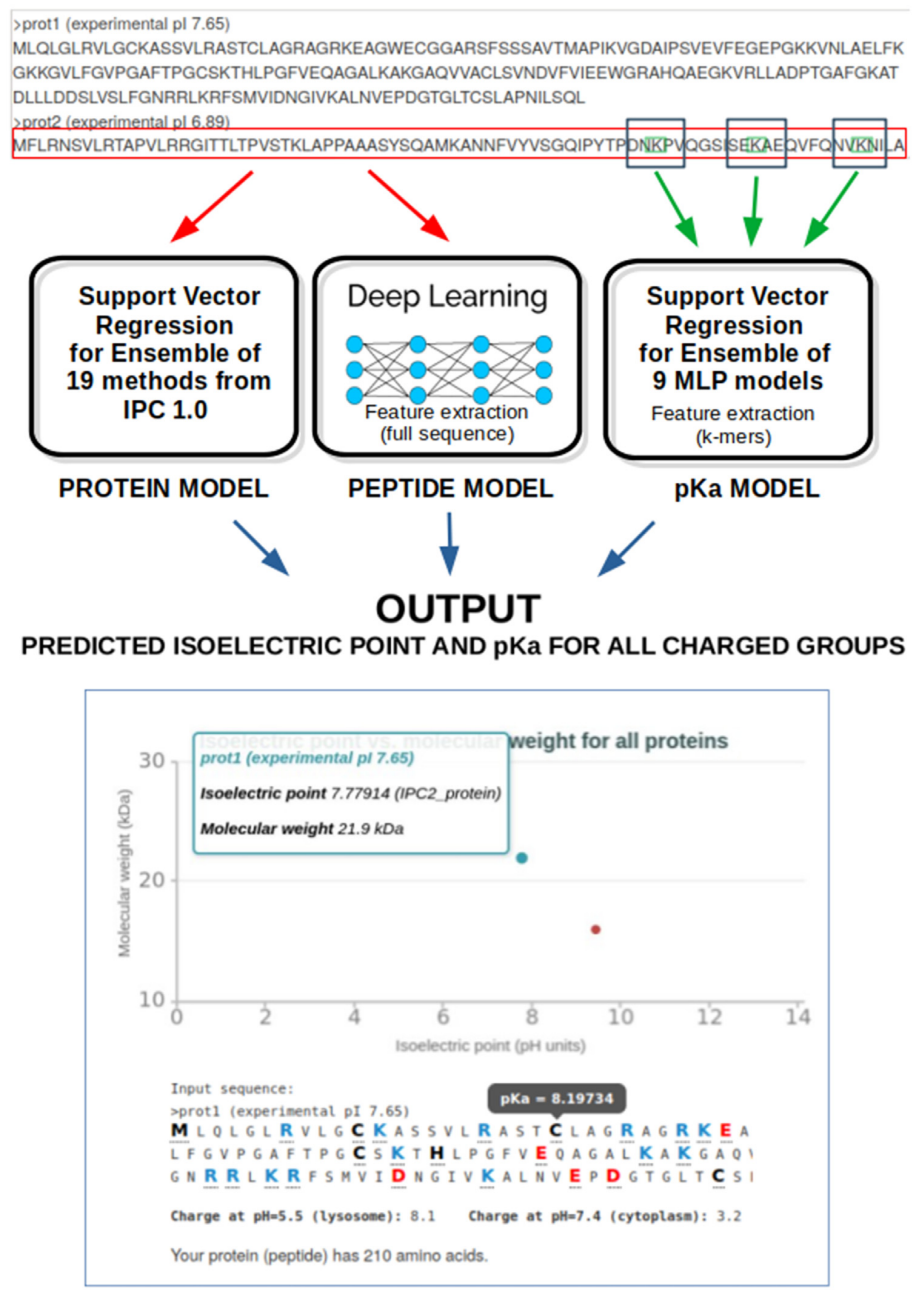
Overview of the IPC 2.0 architecture. The input (amino acid sequence in the plain format or multiple sequences in the FASTA format) is processed by individual machine learning models. Separate models depending the prediction task are used. Isoelectric point prediction for peptides is based on separable convolution model (four channels representing the one-hot-encoded sequence, AAindex features, amino acid counts, and predictions from IPC 1.0). The protein pI and p*K*_a_ prediction models use the ensembles of low level models integrated with support vector regressor. For more details, see Supplementary Figure S1 and ‘Machine Learning Details’ in the Supplementary Material.

In this work, I present a major update of the original IPC algorithm (available at http://isoelectric.org) (12) that significantly extends its capabilities (Figure 1). The IPC 2.0 web server (available at http://www.ipc2-isoelectric-point.org and mirrored at http://ipc2.mimuw.edu.pl) incorporates two major feature upgrades:

- Prediction of the isoelectric point using state-of-the-art machine learning instead of the relatively simple p*K*_a_ optimisation used in IPC 1.0
- Prediction of individual p*K*_a_ values based solely on sequence features

The features were implemented to address the major drawbacks of IPC according to users’ feedback and to improve prediction accuracy. Additionally, a new, larger dataset for peptides was used. The input of IPC 2.0 is the peptide or protein sequence(s) in a one-letter amino acid code (for multiple sequences, the FASTA format is used). For each input sequence, IPC 2.0 runs the machine learning models for the isoelectric point and predicts the p*K*_a_ dissociation constant for each charged residue and the terminal groups of polypeptide chains. Additionally, a scatter plot with the predicted isoelectric points versus the molecular weight is presented for all proteins (in total, output from 21 isoelectric point prediction methods). All the prediction results can be downloaded in CSV format for further analysis.

## MATERIALS AND METHODS

### Datasets

To develop and test the IPC2.0 server, multiple benchmark datasets had been used (Table 1). They can be divided into the following three main sets:

- Protein dataset: contains 2324 non-redundant proteins with experimentally measured isoelectric points (merged data from SWISS-2DPAGE and PIP-DB databases (22,23)). This dataset was also used in IPC 1.0. For more details, see (12).
- Peptide dataset: the datasets from previous studies were merged to form a total of 119 092 non-redundant peptides (6,24,25). Note that this is different peptide dataset than that used for IPC 1.0. For more details, see (12).
- p*K*_a_ dataset: 1337 p*K*_a_ values from 157 proteins were obtained from the PKAD database (17).

**Table 1.** Detailed statistics for the datasets used in IPC 2.0.

Dataset	Entries	Details
**IPC2_protein** IPC_protein_25 (25% test set) IPC_protein_75 (75% training set)	2324 581 1743	The dataset consists of proteins derived from two databases: PIP-DB and SWISS-2DPAGE (22,23). The outliers are defined at 0.5 pH unit difference between the predicted and experimental isoelectric point threshold. The same protein dataset is used in IPC and IPC 2.0. Average protein size: 387 aa.
		
**IPC2_peptide** IPC2_peptide_25 (25% test set) IPC2_peptide_75 (75% training set)	119 092 29 774 89 318	The dataset consists of the peptides from HiRIEF high-resolution isoelectric focusing experiments from Branca et al. 2014 (6) and Johansson et al. 2019 (24). Merged dataset from seven independent experiments: 3.7–4.9 (8,713 peptides), 3.7–4.9 (7361 peptides), 3.7–4.9 (35 595 peptides), 3–10 (23 975), 3–10 (15 000 peptides), 6–11 (36 827 peptides), 6–9 (38 057 peptides). Average peptide size: 14.6 aa.
		
**IPC2_pKa** IPC2_pKa_25 (test set) IPC2_p*K*a_75 (training set)	1337 260 1079	p*K*_a_ values from PKAD database (157 proteins). Due to small number of samples, the test set and training set was built as follows: 260 p*K*_a_ values from 34 proteins used in the pKa Rosetta method (21) were selected as a test set. The remaining samples from the PKAD database were used as the training set.

The full datasets were never used directly. First, the sequences were clustered (to remove duplicates and to average isoelectric point if multiple experimental data existed), then split randomly into 25% and 75% sets (test and training data sets, respectively). The training sets were used for the training and (hyper)parameter optimisation. The test sets were used only once to assess the final performance of the models. For individual datasets’ sequences and experimental isoelectric points, see Supplementary Data 1.

All datasets were clustered to avoid duplicates and, if needed, to merge and average experimental measurements. Next, the datasets were randomly split into 25% testing sets (used only for final benchmarks) and 75% training sets (used for machine learning, hyperparameters optimisation). All presented benchmarks on individual datasets had been calculated with 10-fold cross-validation.

### Feature generation

The most important features for isoelectric point prediction are the sequence itself; the number of charged residues; the amino acid type on the C- and N-termini; and the isoelectric point predicted by simple methods using the Henderson–Hasselbach equation and p*K*_a_ value sets, such as IPC (12), Bjellqvist (26) and DTASelect (27). To engineer additional features, the AAindex—with 566 matrices for 20 standard amino acids—was scanned (28). To select the most informative features, the univariate feature selection with regression (*f_regression*) and mutual information (*mutual_info_regression*) was used (up to 10 highest-scoring features were selected using the *SelectKBest* function from Scikit-learn) (29). For *pK_a_* models, the AAindex scores were calculated using *kmers* of different sizes centred on a charged amino acid (for a pentamer, e.g. xxRxx, this may correspond to ALRWI, GIRAA, WRRIL, etc. For more details, see ‘*Machine Learning Details*’ section in the Supplementary Material). It is important to stress that local protein features, such as secondary structure and solvent accessibility, are valid only for protein sequences. They are irrelevant for short peptides, where, for instance, the use of a mass spectrometer disrupts any higher-order structure of the molecule. Therefore, *pK_a_* predictions should be considered valid only for proteins (>50 amino acids).

### Performance evaluation metrics

The prediction of p*K*_a_ and p*I* values is a regression problem. Therefore, metrics such as root mean square deviation (RMSD), mean absolute error (MAE), Pearson's correlation coefficient (*r*^2^), and the number of outliers were used. While the first three metrics are commonly used, the last must be explained. The outliers were defined at 0.5 and 0.25 pH unit difference thresholds between the predicted and experimental p*I* for proteins and peptides, respectively. Thus, if the prediction disagree with the experimental p*I* by given threshold, such case has been considered as the outlier. The total number of outliers for individual datasets has been used to identify methods returning predictions within reasonable error range.

### Machine learning

In this work, I try to solve three independent problems: the p*I* of proteins, the p*I* of peptides, and the p*K*_a_ of charged residues. Therefore, it is justified to design (at least) three separate models. Any machine learning process begins with the conversion of input data (in this case, polypeptide chains) into a format that can be used by machine learning packages (here, SciPy (30), sklearn (29), Tensorflow (31) and Keras (32)). The simplest approach is to use one-hot encoding, but it should be stressed that this produces a sparse matrix or vector (in this case, it would be *L* × 22, where *L* is the polypeptide length and 22 corresponds to 20 standard amino acid letters plus one for an unknown amino acid and one for padding). If the input varies in length, some padding (peptides; up to 60) or truncation (protein; down to 1000) is inevitable. With regard to p*I* prediction, the number and type of charged groups are most significant; thus, this information (even alone) can be used as the initial vector. Additionally, if possible, the introduction of hand-crafted features is recommended (this is important when the data size is limited). This last step can frequently be omitted because if it is given a sufficiently large dataset, the deep learning approach can learn the features of the model by itself (e.g. the convolution filters for the images). Unfortunately, if data are scarce, the prediction accuracy is hampered, and adding hand-crafted features can be unavoidable to enrich the input vectors with expert knowledge.

In the case considered by this paper, apart from the sequence alone, I used features derived from the sequence (such as charge, length, molecular weight, hydrophobicity, number of charged amino acids, and the predicted p*I* from other methods). The input differs according to the problem to be solved and the machine learning technique used but, in general, the input consists of two major parts: sequence-related and feature-related. Several machine learning approaches were tested. First, I used optimization techniques to find the optimal set of seven p*K*_a_ values for charged residues. In the first version of IPC, basin-hopping with a truncated Newton algorithm (33) was used; here, I used a differential evolution algorithm (34), as it performed significantly better. The population size was set to 50 and all remaining parameters were default (SciPy version (30)). Next, having the initial predictions of p*I* from IPC 1.0, I designed a very simple approach based on support vector regression (SVR) with *RBF* kernel and *GridSearchCV* parameters optimization. The input vector in this case was 19 predicted isoelectric points. Finally, I progressed to more advanced machine learning techniques, namely, deep learning. It is possible to start from simple dense networks (Multi-Layer Perceptron; MLP) with different numbers of dense layers and neurons that are interconnected with dropout and with different activation layers (preferably *selu* and *elu*). The final architecture for peptide isoelectric point prediction benefit from all mentioned information and is based on stacking of separable convolution layers. The input is reshaped in the following way. For peptides, the maximal length is 60 amino acids; thus, this defines the main size of the 2D matrix obtainable after one-hot encoding (all sequences were padded up to 60, if needed). This results in a matrix of 60 × 22 (20 standard amino acids, X for unknown, and 0 for padding). By analogy with the image processing from which convolution has been adopted, this 60 × 22 matrix can be considered as the main image size. Similarly, additional information can be stored in separate channels (for instance an RGB image has three channels for values of red, green, and blue). Here, I used four channels. The first channel was, as stated, a one-hot encoded sequence. The second channel stored information about the most informative features from AAindex. In the third and fourth channels, I encoded the information about 1D features, the information about charged residues counts, and the isoelectric point prediction from IPC 1.0. The input was processed by two separable convolution layers, interconnected with two average pooling layers. The initial kernel size of the filter was set to 22 × 5 to slide across the whole amino acid frame with a window of five amino acids. Then the feature maps were flattened and sent to a standard MLP unit: three dense layers (Supplementary Figure S1).

Machine learning architecture for the prediction of p*K*_a_ is very different, as here the focus is on a single charged amino acid (and its neighbourhood). Therefore, the input is very limited. I decided to use the information related to *kmers* of different size. With increasing size of the *kmer* (from three to 15), we encoded the sequence (one-hot encoding) and the amino acid scores for the most informative features from AAindex. This information was used as input for the MLP unit (three dense layers separated by dropout layer). Next, to boost the performance we used an ensemble of nine models to build final support vector regression model.

In all deep learning models, the ADAM optimiser (35) and hyper-parameter optimisation by RandomizedSearchCV were used. As the optimisation condition, the *mean_squared_error* loss function was used. Apart from the dropout, 10-fold cross-validation and early stopping were used to estimate the robustness of the predictions and to avoid overfitting. During the fitting process, the training set was randomly split (*validation_split* = 0.2). Finally, the performance was estimated for the 25% of cases that had been omitted. For more details, see ‘*Machine Learning Details***’** in the Supplementary Material.

### Other methods

To benchmark IPC 2.0, multiple other methods were compared. The simplest methods of isoelectric point prediction are based on different p*K*_a_ sets and the Henderson–Hasselbach equation (Patrickios (36), Solomon (37), Lehninger (38), EMBOSS (39), Dawson (40), Wikipedia (p*K*_a_ values as presented in Wikipedia page in 2005), Toseland (41), Sillero (42), Thurlkill (16), Rodwell (43), DTASelect (27), Nozaki (44), Grimsley (45), Bjellqvist (26), whose method was implemented as ExPASy ‘Compute p*I*/Mw Tool’ (46), ProMoST (9) and finally IPC 1.0 (12)). Additionally, machine learning methods, such as PredpI (plain, TMT6, iTRAQ8 variants) (6) and pIR (15) were also used. Furthermore, IPC 2.0 p*K*_a_ predictions were compared to those of Rosetta p*K*_a_ (four variants) (21).

### Implementation

The pre-processing, training, and testing of the machine learning models were done in the Python programming language. Among the libraries used, the most important were SciPy (30), sklearn (29), TensorFlow (31), and Keras (32). For the web-server implementation, the Apache server and the PHP programming language were used. In addition, the HTML front-end benefitted from the Twitter Bootstrap and CanvasJS libraries.

## RESULTS

### Isoelectric point prediction

The isoelectric point prediction of the IPC 2.0 method is based on two separate datasets that consist of protein and peptides. The datasets differ in size and in the difficulty of the prediction task. Peptides are much shorter and contain only a small number of charged groups. In contrast, in proteins, multiple additional factors, such as PTMs or solvent accessibility, need to be taken into account. Thus, the estimation of the isoelectric point is much more difficult. The results presented in Table 2 show that the IPC 2.0 models performed the best. Moreover, the more information and more advanced machine learning technique used, the better were the results. The optimisation models (denoted IPC2_protein and IPC2_peptide) use *pKa* sets that are optimal for calculating *pI* with the Henderson-Hasselbach equation (Supplementary Table S1). They perform better than any other methods (RMSD of 0.860 by IPC2_protein versus 0.911 by ProMoST and 0.248 by IPC2_peptide versus 0.405 by Bjellqvist). However, from an machine learning point of view, they are very simple (but at the same time very fast).

**Table 2.** Isoelectric point prediction accuracy on leave-out 25% datasets

Method	Protein dataset^a^				Method	Peptide dataset^b^			
	RMSE	MAE	*R* ^2^	Outliers^c^		RMSE	MAE	*R* ^2^	Outliers^c^
**IPC2.protein.svr.19**	0.8479	0.5906	0.5934	247	**IPC2.peptide.Conv2D**	0.2216	0.1216	0.9761	2691
**IPC2_protein**	0.8608	0.6052	0.5748	251	**IPC2.peptide.svr.19**	0.2299	0.1155	0.9743	2490
IPC_protein	0.8677	0.6109	0.5760	250	**IPC2_peptide**	0.2482	0.1394	0.9700	3179
ProMoST	0.9113	0.6444	0.5183	263	Bjellqvist	0.4051	0.2836	0.9204	11639
Toseland	0.9278	0.6537	0.5095	250	Nozaki	0.4083	0.2673	0.9191	9837
Dawson	0.9365	0.6586	0.4977	263	DTASelect	0.4235	0.2796	0.9130	10606
Bjellqvist	0.9369	0.6536	0.5005	260	Thurlkill	0.4466	0.2535	0.9033	7182
Wikipedia	0.9484	0.6795	0.4860	262	Sillero	0.4747	0.2696	0.8907	7607
Rodwell	0.9579	0.6762	0.4706	262	Dawson	0.4910	0.2642	0.8831	6698
Grimsley	0.9588	0.6953	0.4779	265	Wikipedia	0.5178	0.2974	0.8700	8326
Lehninger	0.9617	0.6783	0.4607	266	Grimsley	0.5264	0.3796	0.8656	15956
Solomon	0.9631	0.6746	0.4606	272	Rodwell	0.5855	0.3429	0.8337	9857
pIR	1.0148	0.7556	0.4161	315	Toseland	0.5860	0.3896	0.8335	13152
Nozaki	1.0164	0.7219	0.3980	288	EMBOSS	0.5971	0.3557	0.8271	11022
Thurlkill	1.0250	0.7573	0.3948	302	PredpI-iTRAQ8	0.6302	0.3503	0.8027	12059
DTASelect	1.0278	0.7798	0.3947	319	PredpI-TMT6	0.6365	0.3518	0.7988	12135
EMBOSS	1.0498	0.7757	0.3734	308	PredpI-plain	0.6480	0.3710	0.7913	12813
Sillero	1.0519	0.7694	0.3461	308	IPC_peptide	0.7459	0.4860	0.7302	13599
Patrickios	2.3764	1.8414	<0	517	Solomon	0.7518	0.4929	0.7259	13777
PredpI-TMT6	NA	NA	NA	NA	Lehninger	0.7697	0.5209	0.7127	15200
PredpI-plain	NA	NA	NA	NA	pIR	0.8529	0.7303	0.6387	27158
PredpI-iTRAQ8	NA	NA	NA	NA	ProMoST	1.1026	0.7562	0.4104	18513
					Patrickios	2.0172	1.3927	<0	22818

^a^Protein dataset consisting of 581 proteins (25% randomly chosen proteins, not used for the training or optimization).

^b^Peptide dataset consisting of 29 774 peptides (25% randomly chosen peptides, not used for the training or optimization).

^c^The outliers were defined at 0.5 and 0.25 pH unit difference between the predicted and experimental p*I* thresholds for the protein and peptide datasets.

*NA*: The PredpI program was designed for peptides only within the 3.7–4.9 pH range; thus, for proteins, it returned 0 and could not be evaluated on the protein dataset.

New machine learning models developed in this study are in **bold**. First version of IPC (12) is underscored. Scores calculated after 10-fold cross-validation. Table is sorted by RMSD. For individual methods’ predictions, see Supplementary Data 2. For more details about the datasets, see Table 1.

The next machine learning approach I used was support vector regression (SVR), in which the isoelectric point predicted by other methods was an input, in a so-called ensemble averaging technique (47,48). The main advantage of SVR is that it has only two parameters (C and gamma for RBF kernel) that need to be optimised. The main disadvantage is that the input features must be already well designed and in a similar space (for instance, enriching the input with more heterogeneous features, such as protein length or molecular weight, prevent the algorithm from converging). The SVR models not surprisingly performed better than the optimisation versions (RMSD of 0.848 and 0.230 for proteins and peptides, respectively). Another key feature of ensemble averaging is that it can be used to reduce the variance of the predictions. This can be seen in this study with the significant reduction of outliers: the IPC2.peptide.svr.19 model produced only 8.3% outliers, while the input methods on average produced ∼32% outliers with only one model with 10.6% outliers.

Finally, it is possible to progress to deep learning techniques in which the amino acid sequence can be used directly by one-hot encoding. Additionally, all remaining hand-crafted features can be added easily (e.g. the most informative features from AAindex; see Supplementary Tables S2–S4). Together with the plethora of ready-to-adapt deep learning architectures, this provides another level of improvement (and training complexity). The final architecture used for peptide *pI* prediction in IPC 2.0 is based on separable convolution kernels scanning multichannel input. The model obtained an RMSD of 0.222. It should be stressed that IPC 2.0 is also a robust method, as it produces the fewest outliers (247 and 2490 for proteins and peptides, respectively). Moreover, due to the strict methodology for clustering and 10-fold cross-validation, the method does not exhibit significant signs of overfitting (compare Table 2 and Supplementary Table S5). In this study, multiple machine learning models were tested (for details, see Supplementary Table S6).

### *pK_a_* dissociation constant prediction

The p*K*_a_ prediction of individual residues is a separate challenge with its own problems that need to be addressed. First, the data are very limited. Moreover, currently available methods (e.g. MCCE, H++ and p*K*_a_ Rosetta) require protein structure and are relatively slow (for instance, the *pK*_a_ Rosetta protocol used for benchmarking in this work requires several hours for a single protein). In contrast, IPC 2.0 uses only sequence-based features and returns results almost instantly with similar accuracy (Table 3 and Supplementary Table S7). The prediction of p*K*_a_ values is based on an SVR ensemble of nine MLP models that use the information derived from *kmers* of different sizes centred on the charged residue. This approach made it possible to capture sequence fingerprints that were located in direct proximity to the charged residue. The overall accuracy of the IPC 2.0 p*K*_a_ prediction was better than that of the p*K*_a_ Rosetta protocol (0.576 versus 0.839), although some p*K*_a_ dissociation constants were clearly worse predicted by IPC 2.0. The main sources of misprediction were His and Tyr residues: for Tyr residues at least, this can be explained by the small number of training points.

**Table 3.** p*K*_a_ prediction accuracy of Rosetta pKa dataset.

Method	Rosetta p*K*_a_ dataset^a^			Method	Rosetta p*K*_a_ dataset^a^		
	RMSE	MAE	Outliers^b^		RMSE	MAE	Outliers^b^
**D** (74; 3.45 ± 0.80)				**Y** (17; 10.89 ± 0.82)			
**IPC2_pKa**	**0.3883**	**0.2238**	**6**	**Rosseta (Site repack)**	**0.7750**	**0.6177**	**7**
Rosseta (Site repack)	0.8193	0.5824	27	Rosseta (Neighbor repack)	0.8370	0.6647	9
Rosseta (Ensemble average)	0.8413	0.5460	25	Rosetta (Standard)	0.9579	0.8000	9
Rosseta (Neighbor repack)	0.8676	0.6378	34	IPC2_pKa	0.9766	0.8261	10
Rosetta (Standard)	1.0651	0.8554	46	Rosseta (Ensemble average)	1.1892	0.9529	13
**H** (76; 6.58 ± 0.98)				**K** (22; 10.66 ± 0.52)			
**Rosseta (Site repack)**	**0.8247**	**0.6408**	**31**	**IPC2_pKa**	**0.2933**	**0.1909**	**2**
IPC2_pKa	0.8523	0.5105	27	Rosseta (Neighbor repack)	0.6216	0.5091	7
Rosseta (Neighbor repack)	0.8559	0.6487	32	Rosetta (Standard)	0.6498	0.5046	8
Rosseta (Ensemble average)	1.0244	0.7566	39	Rosseta (Site repack)	0.6705	0.5227	7
Rosetta (Standard)	1.2303	0.9961	50	Rosseta (Ensemble average)	0.7135	0.5364	6
**E** (71; 4.16 ± 0.80)				**All** (260*)			
**IPC2_pKa**	**0.3625**	**0.1951**	**7**	**IPC2_pKa**	**0.5762**	**0.3364**	**54**
Rosseta (Neighbor repack)	0.8744	0.5887	29	Rosseta (Site repack)	0.8262	0.6165	102
Rosetta (Standard)	0.8880	0.7324	38	Rosseta (Neighbor repack)	0.8332	0.6185	111
Rosseta (Site repack)	0.9303	0.6549	30	Rosseta (Ensemble average)	0.9207	0.6746	114
Rosseta (Ensemble average)	0.9317	0.6972	34	Rosetta (Standard)	1.0300	0.8296	151

^a^For the validation of p*K*_a_, the dataset from Kilambi and Gray (2012) was used (260* residues from 34 proteins). The numbers next to the residue type indicate the number of cases and the average p*K*_a_ value with standard deviation.

^b^The outliers are defined at 0.5 pH unit difference between the predicted and experimental p*K*_a_ threshold.

*The dataset consists of 260 instead of 264 residues due to parsing problems (four missing residues could not be mapped to the protein sequence, due to the wrong residue register). Scores calculated after 10-fold cross-validation.

### IPC 2.0 web server

The IPC 2.0 web server (http://www.ipc2-isoelectric-point.org) takes protein(s) and peptide(s) as input (single sequence or multiple sequences in FASTA format, up to 10 000 residues in total) and returns predictions of p*I* and p*K*_a_ values for individual charged residues. In addition, the outputs are complemented by virtual 2D-PAGE plots. As the server is capable of multiple sequence predictions, its convenient output feature is the CSV format, which can be used for further large-scale analyses (e.g. on the proteome scale 49–52). Additionally, the standalone version of IPC 2.0 is also available on web server site and as Supplementary Data.

## DISCUSSION

In this work, I have presented IPC 2.0, a new web server for isoelectric point and *pKa* dissociation constant prediction based on sequence information only. It uses a state-of-the-art ML methodology and represents an improvement on previous methods. Additionally, IPC 2.0 is the first, fast p*K*_a_ prediction method that can estimate p*K*_a_ values using sequence information alone. It does so within seconds, with a performance level similar to more time-consuming and structure-based methods. To boost the performance of IPC 2.0 and to bypass the limited size of datasets for protein and p*K*_a_ prediction hand-crafted features were used (Supplementary Tables S6 and S7).

However, users should be aware of some of the IPC 2.0 web server's limitations. First, the isoelectric point and p*K*_a_ predictions can be significantly distorted when PTMs are present (e.g. phosphorylation). This should be kept in mind when analysing proteins from eukaryotes that are rich in PTMs (see Supplementary Table S1 in Kozlowski, 2016 [12]). Due to very limited experimental data, it was not possible to develop machine learning models dedicated to PTMs. Another shortcoming of IPC 2.0 is that it only uses sequence information. It is expected that protein structures that contain more information about the charged residue's neighbourhood should improve the prediction performance. Such an approach could help to increase the modest performance of p*K*_a_ predictions.

The high performance of the IPC 2.0 web server (and standalone version) makes it suitable for large-scale analyses related to the charge of proteins and peptides. I believe that biologists will benefit from this web server with its user-friendly interface.

## DATA AVAILABILITY

IPC 2.0 (http://www.ipc2-isoelectric-point.org), including the web service, datasets, standalone scripts, and documentation, has been donated to the public domain. Therefore, it can be freely used for any legal purpose. Nevertheless, the machine learning libraries used by some models are: sklearn, Tensorflow and Keras, which are under BSD, Apache and MIT licences, respectively. The web server will be available at the given web address for at least 10 years.

## Supplementary Material

gkab295_Supplemental_Files
