# Supplementary material for: IPC 2.0: prediction of isoelectric point and pKa dissociation constants
Source: Nucleic Acids Res. 2021 Apr 27;49(W1):W285–92. doi: 10.1093/nar/gkab295 (PMC8262712; doi:10.1093/nar/gkab295)
Supplement: gkab295_Supplemental_Files [file gkab295_supplemental_files.zip › NAR-IPC2-supplement.pdf]

Supplementary Materials for

IPC 2.0 – prediction of isoelectric point and pK_a dissociation constants

Lukasz P. Kozlowski

Institute of Informatics, Faculty of Mathematics, Informatics, and Mechanics, University of Warsaw, Warsaw, Mazovian Voivodeship, 02-097, Poland

Correspondence to: lukasz.kozlowski.lpk@gmail.com

This PDF file includes:

“Machine Learning Details” section

Supplementary Tables 1-7

Supplementary Figure 1

Captions for Supplementary Data 1-4

Machine Learning Details

Deep Separable Convolutional Neural Network Based Regression

The input of the convolution model predicting isoelectric points for peptides has the shape of 60x22x4. The number of channels is four. The size of the 'image' is defined by the input amino acid sequence. The maximal length for peptides is set to 60 and if the input is shorter, it is padded to 60. The sequence is one-hot-encoded (20 standard amino acids, X for unknown, and 0 for padding). This gives the 60 x 22 matrix as the base shape of the 'image'. This is the first channel. Next is a channel with information derived from the most informative features from AAindex. In the case of the peptides, there are 15 features (Supplementary Table 2), and the remaining seven rows are padded. In the next two channels are stored information about 1D features (amino acid counts and predictions from IPC 1.0). The scalars are duplicated to form a 60-long vector (this made it possible to pass the information about these features to multiple filters; Supplementary Figure 1a).

Now, as the input has been transferred into a suitable format, the convolution layers can be used. First, separable convolution is used to separate the features from different channels. Next, *AveragePooling* is used. Finally, the information from all filters was aggregated (flattened) and passed to three dense layers with 60 neurons and a final dense neuron (Supplementary Figure 1b). Some parameters (e.g. filter size, activation, number of layers) were optimised by RandomizedSearchCV. To a limited extent, the presented architecture was compared with deeper models (e.g. VVG-like, Inception-like, or with layers, such as MaxPooling2D, Dropout, BatchNormalization) without showing much improvement. Additionally, the information in channels 1 and 2 is arranged by amino acid, and the information in channels 3 and 4 is arranged by whole sequence. Thus, it seems logical that the architecture could be separated into two sub-models, in which the features from channels 3 and 4, instead of being duplicated, are processed by a dedicated MLP sub-model and then concatenated with a convolution sub-model for channels 1 and 2. However, such architecture performed worse than the four-channel, separable convolution model used as the final architecture.

Multilayer Perceptron Ensemble Support Vector Regression (MLP-SVR)

One of the major boosts in performance resulted from the use of support vector regression on the ensemble of IPC 1.0 methods. It worked very well as a robust averaging technique, limiting the number of outliers (compare, for instance, *IPC2.peptide.SVR19* with *IPC2_peptide* – optimisation-based model). Therefore, an interesting experiment was to use a similar approach for pK_a prediction. The only problem was that we lacked a sequence-based method for pK_a prediction. To generate an ensemble of pK_a prediction methods I used an iterative approach. I developed simple MLP models (dense, dropout, dense, dense) learned on the *kmers* focused on the given charged amino acid (with length 3, 5, 7, 9, 11, 13, 15): longer *kmers* did not improve the prediction accuracy. The first seven models used only the sequence information (one-hot encoding, the input of 3*21, 5*21, ...). The next

five predictors (*kmers*: 3, 5, 7, 9, 11) used the most informative features from AAindex: longer *kmers* did not improve the prediction accuracy (the input of 3*20, 5*20, ...). Next, I combined these two sources of information (another five models with the same *kmer* size of 3, 5, 7, 9, 11 resulted in the input of 3*(21+20), 5*(21*20), …): longer *kmers* did not improve the prediction accuracy. Based on the performance, I additionally developed an additional mixed model (13mer from sequence and 5mer from AAindex). In consequence, I obtained 18 MLP models. To speed up the run-time (loading into memory multiple DL models has a huge computational overhead), I iteratively dropped MLP models from the worst-performing until this did not harm prediction accuracy significantly. In consequence, an ensemble of nine MPL models was chosen to form the input for the SVR model. It should be stressed that even a trimer sequence alone is informative, and integrating the information from multiple MPL models improved the prediction accuracy significantly (different *kmers* from sequences in one-hot encoding, and the features from AAindex). For performance statistics see Supplementary Table S7.

Supplementary Table S1. *The optimised dissociation constant (pK_a) of charged groups of seven amino acids and NH^+ and COO^- groups. IPC_peptide/IPC_protein refers to the work from 2016. New pK_a sets were found using differential evolution (instead of basin-hopping). Interestingly, the IPC2_protein set neglected the importance of the termini charges for the overall isoelectric point calculation of long molecules.*

	**NH^+**	**COO^-**	**Asp**	**Glu**	**Cys**	**Tyr**	**His**	**Lys**	**Arg**
IPC_protein	*9.094*	2.869	3.872	4.412	7.555	10.850	5.637	9.052	11.840
**IPC2_protein**	*5.779*	6.065	3.766	4.497	7.890	11.491	5.492	9.247	10.223
IPC_peptide	*9.564*	2.383	3.887	4.317	8.297	10.071	6.018	10.517	12.503
**IPC2_peptide**	*7.947*	*2.977*	3.969	4.507	9.454	9.153	6.439	8.165	11.493

Supplementary Table S2. The most informative AAindex features for peptide isoelectric point prediction (IPC2_peptide_75 dataset)

	Score	Aaindex	Description
1	100524	FAUJ880112	Negative charge (Fauchere et al.; 1988)
2	26404	CHAM830107	A parameter of charge transfer capability (Charton-Charton; 1983)
3	23089	ZIMJ680103	Polarity (Zimmerman et al.; 1968)
4	21499	RICJ880106	Relative preference value at N3 (Richardson-Richardson; 1988)
5	20510	FAUJ880110	Number of full nonbonding orbitals (Fauchere et al.; 1988)
6	19881	HOPA770101	Hydration number (Hopfinger; 1971); Cited by Charton-Charton (1982)
7	15707	ISOY800107	Normalized relative frequency of double bend (Isogai et al.; 1980)
8	13141	RICJ880105	Relative preference value at N2 (Richardson-Richardson; 1988)
9	12119	ENGD860101	Hydrophobicity index (Engelman et al.; 1986)
10	12106	PRAM900101	Hydrophobicity (Prabhakaran; 1990)
11	0.853	MITS020101	Amphiphilicity index (Mitaku et al.; 2002)
	0.761	ZIMJ680103	Polarity (Zimmerman et al.; 1968)
	0.747	FAUJ880112	Negative charge (Fauchere et al.; 1988)
12	0.283	FINA910102	Helix initiation parameter at posision i;i+1;i+2 (Finkelstein et al.; 1991)
13	0.269	FINA910104	Helix termination parameter at posision j+1 (Finkelstein et al.; 1991)
14	0.269	KARS160120	Weighted minimum eigenvalue based on the atomic numbers (Karkbara-Knisley; 2016)
15	0.269	FAUJ880111	Positive charge (Fauchere et al.; 1988)
	0.252	CHAM830107	A parameter of charge transfer capability (Charton-Charton; 1983)
	0.245	HOPA770101	Hydration number (Hopfinger; 1971); Cited by Charton-Charton (1982)
	0.232	FAUJ880110	Number of full nonbonding orbitals (Fauchere et al.; 1988)

Only a training set (75% of the data; 89,319 peptides) was used. The results for univariate feature selection with regression (f_regression) and mutual information (mutual_info_regression) are presented (F-values and mutual information scores, respectively). First, up to 10 highest-scoring features for each criterion were selected, using the SelectKBest function from Scikit-learn. Next, their correlation and importance to each other was assessed by recursive feature elimination (RFE) with a linear regression estimator. The grey lines correspond to the features either doubled by f_regression/mutual_info_regression or dropped by RFE.

Supplementary Table S3. The most informative AAindex features for protein isoelectric point prediction (IPC2_protein_75 dataset)

	Score	AAindex	Description
1	49.296	FAUJ880112	Negative charge (Fauchere et al.; 1988)
2	26.360	CHAM830107	A parameter of charge transfer capability (Charton-Charton; 1983)
3	21.992	ROBB760102	Information measure for N-terminal helix (Robson-Suzuki; 1976)
4	19.906	RICJ880106	Relative preference value at N3 (Richardson-Richardson; 1988)
5	18.236	SUEM840102	Zimm-Bragg parameter sigma x 1.0E4 (Sueki et al.; 1984)
6	17.708	CHOP780204	Normalized frequency of N-terminal helix (Chou-Fasman; 1978b)
7	17.357	FAUJ880110	Number of full nonbonding orbitals (Fauchere et al.; 1988)
8	16.824	RICJ880105	Relative preference value at N2 (Richardson-Richardson; 1988)
9	16.492	QIAN880103	Weights for alpha-helix at the window position of -4 (Qian-Sejnowski; 1988)
10	16.460	FAUJ880108	Localized electrical effect (Fauchere et al.; 1988)
11	0.093	NISK860101	14 A contact number (Nishikawa-Ooi; 1986)
12	0.093	QIAN880123	Weights for beta-sheet at the window position of 3 (Qian-Sejnowski; 1988)
	0.092	FAUJ880112	Negative charge (Fauchere et al.; 1988)
13	0.090	LEVM760104	Side chain torsion angle phi(AAAR) (Levitt; 1976)
14	0.086	QIAN880119	Weights for beta-sheet at the window position of -1 (Qian-Sejnowski; 1988)
15	0.085	NAKH920107	AA composition of EXT of multi-spanning proteins (Nakashima-Nishikawa; 1992)
16	0.083	JUKT750101	Amino acid distribution (Jukes et al.; 1975)
17	0.083	CASG920101	Hydrophobicity scale from native protein structures (Casari-Sippl; 1992)
18	0.082	CORJ870107	TOTFT index (Cornette et al.; 1987)
19	0.082	WILM950102	Hydrophobicity coefficient in RP-HPLC; C8 with 0.1%TFA/MeCN/H2O (Wilce et al. 1995)

*Only the training set (IPC_protein_75 dataset, 75% of the data; 1,743 proteins) was used. The results for univariate feature selection with regression (f_regression) and mutual information (mutual_info_regression) are presented (F-values and mutual information scores, respectively). First, up to 10 highest-scoring features for each criterion were selected, using the **SelectKBest** function from Scikit-learn. Next, their correlation and importance were assessed by recursive feature elimination (**RFE**) with a linear regression estimator. The grey lines correspond to the features either doubled by f_regression/mutual_info_regression or dropped by RFE.*

Supplementary Table S4 *The most informative AAindex features for pK_a prediction (IPC2_pKa_75 dataset)*

	Score	Aaindex	Description
1	267.214	FINA910101	Helix initiation parameter at posision i-1 (Finkelstein et al.; 1991)
2	257.050	KLEP840101	Net charge (Klein et al.; 1984)
3	237.666	QIAN880113	Weights for alpha-helix at the window position of 6 (Qian-Sejnowski; 1988)
4	232.743	ZIMJ680104	Isoelectric point (Zimmerman et al.; 1968)
5	200.743	AURR980104	Normalized positional residue frequency at helix termini N'(Aurora-Rose; 1998)
6	193.443	FAUJ880112	Negative charge (Fauchere et al.; 1988)
7	185.169	FAUJ880108	Localized electrical effect (Fauchere et al.; 1988)
8	182.919	RICJ880105	Relative preference value at N2 (Richardson-Richardson; 1988)
9	177.771	QIAN880129	Weights for coil at the window position of -4 (Qian-Sejnowski; 1988)
10	176.638	QIAN880111	Weights for alpha-helix at the window position of 4 (Qian-Sejnowski; 1988)
11	1.508	FASG760103	Optical rotation (Fasman; 1976)
12	1.506	NAKH900111	Transmembrane regions of non-mt-proteins (Nakashima et al.; 1990)
13	1.485	ISOY800107	Normalized relative frequency of double bend (Isogai et al.; 1980)
14	1.484	BIGC670101	Residue volume (Bigelow; 1967)
15	1.476	FUKS010101	Surface composition of amino acids in intracellular proteins of thermophiles (percent) (Fukuchi-Nishikawa; 2001)
16	1.476	OOBM850102	Optimized propensity to form reverse turn (Oobatake et al.; 1985)
17	1.475	OOBM850101	Optimized beta-structure-coil equilibrium constant (Oobatake et al.; 1985)
18	1.474	HUTJ700102	Absolute entropy (Hutchens; 1970)
19	1.473	HUTJ700103	Entropy of formation (Hutchens; 1970)
20	1.470	CORJ870108	TOTLS index (Cornette et al.; 1987)

The 1,079 pentamers centred on charged residue are used. The results for univariate feature selection with regression (f_regression) and mutual information (mutual_info_regression) are presented (F-values and mutual information scores, respectively). First, up to 10 highest-scoring features for each criterion was selected, using the SelectKBest function from Scikit-learn. Next, their correlation and importance to each other were assessed by recursive feature elimination (RFE) with a linear regression estimator.

Supplementary Table S5. *Isoelectric point prediction accuracy on training datasets (with 10-fold cross-validation)*

Method	Protein dataset[a]				Method	Peptide dataset[b]			
	RMSE	MAE	R^2	Outliers[c]		RMSE	MAE	R^2	Outliers[c]
**IPC2.protein.svr.19**	0.8217	0.5664	0.6203	685	**IPC2.peptide.Conv2D**	0.2252	0.1224	0.9755	8071
**IPC2_protein**	0.8231	0.5785	0.6166	718	**IPC2.peptide.svr.19**	0.2341	0.1163	0.9735	7547
<u>IPC_protein</u>	0.8355	0.5875	0.6048	717	**IPC2_peptide**	0.2528	0.1402	0.9691	9540
Toseland	0.8961	0.6390	0.5471	770	Bjellqvist	0.4086	0.2857	0.9194	34913
ProMoST	0.8973	0.6334	0.5447	795	Nozaki	0.4137	0.2700	0.9173	29782
Dawson	0.9181	0.6431	0.5193	772	DTASelect	0.4306	0.2836	0.9104	32071
Bjellqvist	0.9206	0.6373	0.5208	783	Thurlkill	0.4542	0.2580	0.9003	21763
Wikipedia	0.9284	0.6650	0.5128	761	Sillero	0.4832	0.2750	0.8872	23072
Rodwell	0.9357	0.6619	0.5046	768	Dawson	0.4990	0.2691	0.8797	20104
Grimsley	0.9366	0.6848	0.5030	808	Wikipedia	0.5258	0.3031	0.8664	25115
Solomon	0.9440	0.6581	0.4953	772	Grimsley	0.5308	0.3821	0.8639	47680
Lehninger	0.9447	0.6629	0.4955	761	Rodwell	0.5945	0.3481	0.8293	29511
Nozaki	1.0032	0.7104	0.4279	845	Toseland	0.5954	0.3955	0.8288	39597
DTASelect	1.0153	0.7681	0.4148	942	EMBOSS	0.6081	0.3640	0.8214	33805
Thurlkill	1.0157	0.7500	0.4175	893	PredpI-iTRAQ8	0.6270	0.3484	0.8059	36035
pIR	1.0360	0.7652	0.3929	965	PredpI-TMT6	0.6331	0.3497	0.8021	36251
EMBOSS	1.0377	0.7630	0.3905	884	PredpI-plain	0.6441	0.3693	0.7951	38232
Sillero	1.0404	0.7562	0.38837	891	<u>IPC_peptide</u>	0.7573	0.4936	0.7230	41003
Patrickios	2.2315	1.7492	<0	1555	Solomon	0.7632	0.5006	0.7186	41500
PredpI-iTRAQ8	NA	NA	NA	NA	Lehninger	0.7813	0.5286	0.7052	45756
PredpI-TMT6	NA	NA	NA	NA	pIR	0.8449	0.7251	0.6476	81129
PredpI-plain	NA	NA	NA	NA	ProMoST	1.1134	0.7647	0.4012	55884
					Patrickios	2.0328	1.4069	<0	68635

[a] *Protein dataset consisting of 1,743 proteins (75% randomly chosen proteins, used for the training and hyperparameter optimisation).*

[b] *Peptide dataset consisting of 89,319 peptides (75% randomly chosen peptides, used for the training and hyperparameter optimisation).*

[c] *The outliers are defined at 0.5 and 0.25 pH unit difference between the predicted and experimental pI threshold for the protein and peptide datasets.*

NA: The PredpI program was designed for peptides only within the 3.7–4.9 pH range; thus, for proteins, it returned 0 and could not be evaluated on the protein dataset.

The machnie learning models developed in this study are in ***bold.*** *First version of IPC (Kozlowski, 2016) are <u>underscored</u>. Table is sorted by RMSD. For individual methods' predictions, see Supplementary Data 2. For more details about the datasets, see Table 1.*

Supplementary Table S6. *The effect of model architecture on the performance of isoelectric point prediction*

Method	Peptide test dataset (30,279)				Method	Protein test dataset (581)			
	RMSE	MAE	R^2	Outliers		RMSE	MAE	R^2	Outliers
IPC2.peptide.Conv2D	0.2216	0.1216	0.9761	2691	IPC2.protein.svr.19	0.8466	0.5907	0.5965	247
IPC2.peptide.svr.19	0.2298	0.1155	0.9743	2490	IPC2_protein	0.8590	0.6052	0.5835	251
IPC2.peptide19	0.2376	0.1271	0.9726	2980	IPC_protein	0.8679	0.6109	0.5779	250
IPC2.peptide1320	0.2394	0.1245	0.9721	3055	ProMoST	0.9116	0.6443	0.5219	263
IPC2_peptide	0.2483	0.1394	0.9700	3179					
Bjellqvist	0.4051	0.2836	0.9204	11639					
IPC_peptide	0.7458	0.4860	0.7302	13599					

IPC2.peptide.Conv2D – an input layer is an 'image' (60x22x4). The rows correspond to amino acid register (up to 60, padded if necessary). The columns in the first channel correspond to amino acid sequence (60 x 22: 20 standard, X for unknown amino acid and 0 for padding). The second channel corresponds to the most informative features from AAindex (60 x 15). The third channel contains charged amino acid counts and in the fourth channel the predicted pI *from other simple methods is stored (60 x 20). The scalars in the third and fourth channels were duplicated to form a 60-long vector. Then, the SeparableConvolution2D, AveragePooling2D, and Dense layers follow. For details of the model architecture see Supplementary Figure S1.*

IPC2.peptide.svr.19 – a support vector regression model with 19 isoelectric points predicted by simple methods (those that use the Henderson-Hasselbach equation; including IPC2_peptide model). The input was limited to pI values only, as adding other features worsened the SVR convergence and the prediction accuracy. SVR parameters were optimised by GridSearchCV (RBF kernel, C = 1,500, epsilon = 0.1293). Note that this model is better than the optimised version (IPC2_peptide), which means that SVR could learn from pI predicted by other methods better than the optimisation and even better than simple MLP models based on sequence alone (IPC2.peptide1320) – the same input as IPC2.peptide19. Additionally, the SVR model produces the fewest outliers.

IPC2.peptide19 – a model that as input takes 19 isoelectric points predicted by simple methods (the same input as IPC2.peptide.svr.19). Multi-Layer Perceptron (MLP) model: dense (760, selu), dense (760, softplus), dense(190, selu), dense(1). The number of neurons and type of activation was optimised by RandomizedSearchCV.

IPC2.peptide1320 – a model that as input takes one-hot-encoded sequence (a flat vector of 1320; 60x22). Multi-Layer Perceptron (MLP) model: dense (1320, softplus), dropout (0.7), dense (60, selu), dense (30, selu), dense (1). The number of neurons and type of activation was optimised by RandomizedSearchCV.

IPC2_peptide – a simple model based on the optimisation of pK_a *values done similarly as in 2016 (IPC 1.0), but this time using a bigger and more robust dataset (119,093 peptides, split into 75% for training and 25% for validation) and differential_evolution instead of basin-hopping. For individual* pK_a *values check Supplementary Table S1.*

Bjellqvist – the best method on peptide dataset developed by others (based on a simple algorithm using pK_a *values by Bjellqvist and the Henderson-Hasselbach equation; used in the Expasy Compute pI/MW tool)*

IPC_peptide – a peptide model based on basin-hopping optimisation of pK_a *values performed in 2016 (IPC 1.0)*

IPC2.protein.svr.19 – identical to IPC2.peptide.svr.19, but optimised with the protein dataset

IPC2_protein – identical to IPC2_peptide, but optimised with the protein dataset

IPC_peptide – a peptide model based on basin-hopping optimisation of pK_a *values done in 2016 (IPC 1.0)*

ProMoST – the best method for protein dataset developed by others (based on a simple algorithm using pK_a *values and the Henderson-Hasselbach equation; 72-parameter model including C- and N-termini corrections for charges)*

Supplementary Table S7. *Overall pK_a prediction accuracy of all models on Rosetta pKa dataset (260 charged residues)*

	RMSD	MAE	R^2	Outliers
**IPC2.mlp-svr.9**	**0.5684**	**0.5684**	**0.9431**	**54**
IPC2.seq7.aaIndex7*	0.6609	0.6609	0.9246	81
IPC2.seq13*	0.6708	0.6708	0.9200	68
IPC2.seq11*	0.6775	0.6775	0.9200	69
IPC2.seq7*	0.6777	0.6777	0.9213	73
IPC2.seq5.aaIndex5*	0.6827	0.6827	0.9184	73
IPC2.seq9*	0.7092	0.7092	0.9150	74
IPC2.seq5*	0.7387	0.7387	0.9014	75
IPC2.seq15*	0.7468	0.7468	0.9015	84
IPC2.seq3.aaIndex3*	0.7807	0.7807	0.8945	94
IPC2.aaIndex5	0.7865	0.7865	0.8946	103
IPC2.aaIndex3	0.8145	0.8145	0.8871	115
IPC2.seq9.aaIndex9	0.8236	0.8236	0.8835	79
**Rosseta (Site repack)**	0.8254	0.8254	0.8776	102
IPC2.seq13.aaIndex5	0.8298	0.8298	0.8732	67
**Rosseta (Neighbor repack)**	0.8305	0.8305	0.8847	111
IPC2.seq3	0.8463	0.8463	0.8784	96
**Rosseta (Ensemble average)**	0.9138	0.9138	0.8575	114
IPC2.seq11.aaIndex11	0.9142	0.9142	0.8516	79
IPC2.aaIndex7	0.9974	0.9974	0.8343	101
IPC2.aaIndex9	1.0257	1.0257	0.8119	75
**Rosetta (Standard)**	1.0285	1.0285	0.8265	151
IPC2.aaIndex11	1.0946	1.0946	0.7927	78

IPC2.mlp-svr.9 – a Multilayer Perceptron Ensemble Support Vector Regression model (MLP-SVR). As an input, uses nine best-performing MLP models (asterisk).

*IPC2.seq(kmer) – MLP model that takes as an input flat vector of kmer, centred on charged residue – sequence in one-hot-encoding format (for instance, for IPC2.peptide.seq3 the input is 3*21=63 [L]). Multi-Layer Perceptron (MLP) model: dense (L*10/L*8/L*6, selu/elu), dropout (0.7), dense (L*8/L*4/L*3,* selu/elu*), dense (L*2/L/L,* selu/elu*), dense(1). To limit the number of parameters, the longer the kmer used, the smaller the multiplier in dense layers (for instance, in kmers of three, it was L*10, L*8, L*2, and in kmers of 15, it was L*6, L*3, L). To limit overfit, a very high dropout level has been used.*

*IPC2.AAindex(kmer) – MLP model that takes as an input a flat vector of the 20 most informative AAindex features for the kmer centred on charged residue (for instance, for IPC2.AAindex3 the input is 3*20=60 [L])*

IPC2.seq(kmer).AAindex(kmer) – MLP model for which the input is derived from the sequence and AAindex features (kmers can be different sizes).

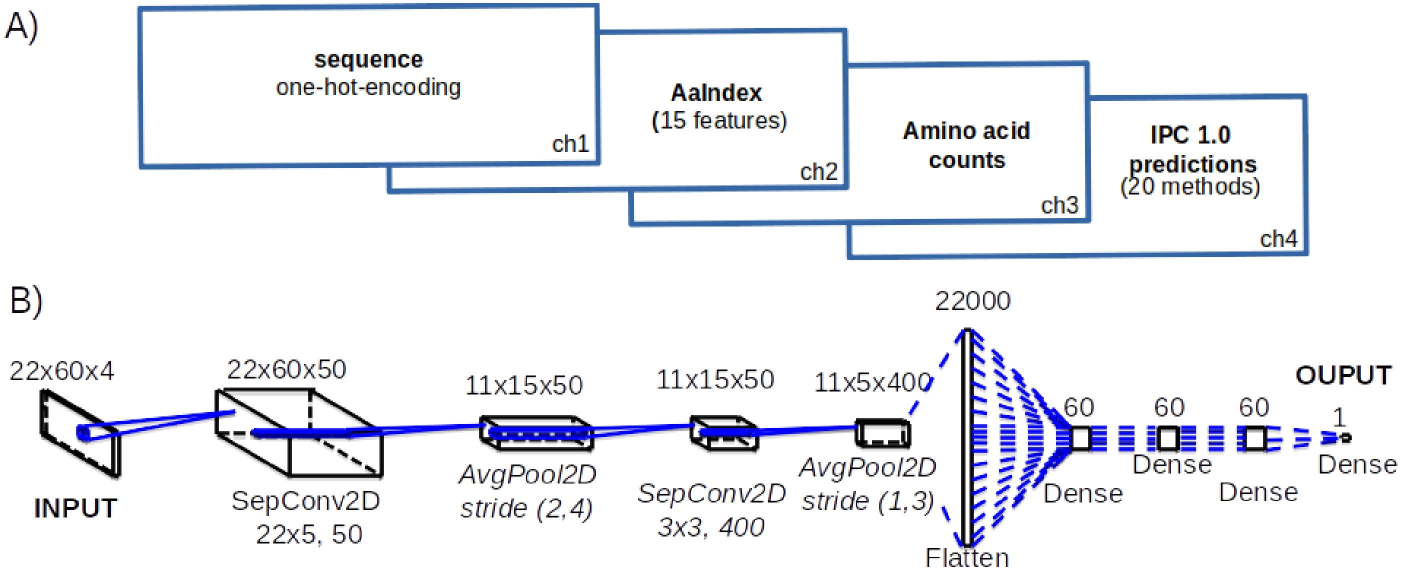

Supplementary Figure S1. *Deep learning architecture for peptide isoelectric point prediction. A) the input is integrated as a four-channel 'image'. The length of the peptide is up to 60 amino acids (with padding if needed; the width). The are 20 amino acids plus X for unknown and 0 for padding, giving 22 in total. In the first channel, the sequence in one-hot-encoding format is stored. In the second channel, are the AAindex features (for details, see Supplementary Table S2; 15 rows, seven remaining padded). Both channels store the information by amino acid. In the third and fourth channels, the amino acid counts and IPC 1.0 predictions are stored. The scalars in those two channels are extended into vectors (an individual row corresponds to a single prediction, e.g. prediction by Dawson*60). This makes it possible to share the information about IPC 1.0 predictions across many filters during the convolution. B) the input is processed by separable convolution filters (the use of separatable filters is crucial) followed by average pooling (here, better than maxpooling). The initial filters have a size of 22 x 5 to allow efficient amino-acid-related motif discovery in the first and second channels. After two rounds of convolution and pooling, everything is flattened and processed by three dense layers. In all layers selu activation was used.*

Supplementary Data 1. The file contains the protein, peptide, and pK_a datasets used for the training and testing of IPC 2.0. The files *_25.txt contain randomly selected test sets. The files *_75.txt contain randomly selected training sets. The files *_100.txt denote complete sets (not used directly).

Webserver site: http://ipc2.mimuw.edu.pl/s1_datasets.7z

RepOD repository: https://doi.org/10.18150/QLPZDQ

Supplementary Data 2. The file contains the ML models (PICKLE files for SVR from Sklearn and HDF5 and JSON files for DL from Keras and Tensorflow)

Webserver site: http://ipc2.mimuw.edu.pl/s2_models.7z

RepOD repository: https://doi.org/10.18150/34GBOB

Supplementary Data 3. The file contains the protein, peptide, and pK_a predictions for all methods

Webserver site: http://ipc2.mimuw.edu.pl/s3_predictions.7z

RepOD repository: https://doi.org/10.18150/CAG3QJ

Supplementary Data 4. The file contains IPC2 standalone version

Webserver site: http://ipc2.mimuw.edu.pl/s4_IPC2_standalone.7z

RepOD repository: https://doi.org/10.18150/7LDND3
